# Distribution of Lipoprotein(a) Levels and Clinical Associations in a Lebanese Adult Population: A Retrospective Observational Study

**DOI:** 10.3390/jcm15041461

**Published:** 2026-02-13

**Authors:** Alaaeddine El Ghazawi, Mahmoud Hammad, Zyad Saifi, Sarah Omran, Samir Alam, Marwan M. Refaat

**Affiliations:** 1 Cardiology Division, Department of Internal Medicine, American University of Beirut Medical Center, Beirut P.O. Box 11-0236, Lebanon; 2 Faculty of Medicine, American University of Beirut, Beirut P.O. Box 11-0236, Lebanonsmo08@mail.aub.edu (S.O.); 3 Department of Biochemistry and Molecular Genetics, Faculty of Medicine, American University of Beirut, Beirut P.O. Box 11-0236, Lebanon

**Keywords:** lipoprotein, Lp(a), Middle East, Lebanon, adult, cardiovascular diseases

## Abstract

**Background**: Lipoprotein(a) (Lp(a)) is a genetically determined lipid particle associated with atherosclerotic cardiovascular disease. Despite growing evidence supporting the clinical relevance of Lp(a) in cardiovascular risk stratification and the emergence of potential therapies targeting elevated Lp(a) levels, Lp(a) testing remains underutilized, with reported rates below 20–30%. This study aims to explore Lp(a) levels in the Lebanese population and their association with the vascular and metabolic burden of diseases. **Methods**: We conducted a retrospective observational study of patients who underwent Lp(a) level testing at the American University of Beirut Medical Center between 2010 and 2023. Data were extracted using the EPIC electronic medical record system, and statistical analyses were performed using IBM SPSS Statistics Version 28. **Results**: This study included 456 patients; the mean age was 50 ± 13, and the mean Lp(a) level was 25 ± 28 mg/dL. Mean Lp(a) was higher in females than in males (28 ± 32 mg/dL versus 23 ± 25 mg/dL), and 25.9%, 12.9%, and 7.6% of the population had Lp(a) levels ≥ 30, ≥50, and ≥70 mg/dL respectively. Logistic regression analysis showed no significant association between Lp(a) levels and cardiovascular factors including dyslipidemia, hypertension, coronary artery disease, previous coronary artery bypass graft, and previous myocardial infarction. Similarly, no significant correlation was found between Lp(a) and LDL, HDL, total cholesterol, triglyceride, and HbA1c. Subgroup analysis showed a significant relationship between Lp(a) levels > 50 mg/dL and atrial fibrillation. **Conclusions**: This study explores the distribution of Lp(a) levels in a Middle Eastern tertiary-care population and provides population-specific descriptive data, addressing an important gap in the existing literature.

## 1. Introduction

Lipoprotein(a) (Lp(a)) is a combination of a low-density lipoprotein (LDL) particle and the apolipoprotein(a) connected noncovalently and through a disulfide bond [1]. Lp(a) plasma levels are primarily genetically determined, with approximately 90% determined by genetic factors [2,3]. Interindividual variability in Lp(a) levels is primarily driven by polymorphisms in the Lp(a) gene, particularly variations in the number of kringle IV2 (KIV2) repeats within apolipoprotein(a). A lower number of KIV2 repeats is associated with higher plasma Lp(a) concentrations [3]. Lp(a) levels can be measured and reported in mass (mg/dL) or molar concentration (nmol/L) [4].

Studies have shown an association between Lp(a) levels and atherosclerotic cardiovascular disease (ASCVD) [1,4]. Elevated Lp(a) serum levels were correlated with both premature and advanced forms of atherosclerosis, ischemic heart conditions, and aortic calcification [5], in a dose–response relationship [6]. Indeed, patients with a subtle increase in Lp(a) serum levels may experience a significant increase in long-term cardiovascular risk [7]. The pathophysiology behind Lp(a) levels increasing the risk of these diseases was hypothesized to be through promoting thrombosis and inflammation [1,7]. Lp(a) has been suggested to increase the cellular cholesterol loading capacity (CLC), whereas lipoprotein apheresis caused a drop in Lp(a) and a subsequent decrease in CLC [8]. This increase in CLC can increase the atherogenicity and thus impose a higher risk of ASCVD.

In addition to ASCVD, increased Lp(a) levels were associated with a higher risk of arrhythmias, most notably, atrial fibrillation (AF), in multiple studies [7,9]. The pro-atherogenic and proinflammatory pathophysiologic properties discussed above could explain this association [7].

Moreover, the pro-thrombotic features associated with Lp(a) increase the likelihood of thromboembolic events in individuals with AF, complicating disease management further [1,7]. Lp(a) levels could have a more influential role in certain individuals. For instance, patients with chronic kidney disease (CKD) frequently show diminished clearance of Lp(a) [10,11]. This heightens the likelihood of cardiovascular diseases and fatalities. In addition, patients with diabetes also undergo certain changes in Lp(a) metabolism. Some studies report that diabetes can decrease Lp(a) levels, though the exact mechanism remains unclear [12].

Although elevated Lp(a) levels were associated with a higher risk of various cardiovascular diseases, there has been no clear threshold above which Lp(a) levels are considered pathological or require intervention [4]. This could be attributed to genetic factors along with ethnic variability [4]. Limited research has been done to assess Lp(a) levels in the Middle East region, with most studies tackling its role in the Western communities. Accordingly, we endeavored to study the Lp(a) characteristics and associated outcomes in the Lebanese population, as a representative of the Middle East region. Our study was conducted in a large tertiary-care referral center in Lebanon, the American University of Beirut Medical Center (AUBMC).

This study aims to assess the level distribution of Lp(a) and to evaluate the associations between Lp(a) levels and cardiovascular and metabolic variables among Lebanese patients who underwent Lp(a) testing between 2013 and 2023 at the AUBMC. We hypothesized that the Lp(a) level distribution and associations may vary from those reported in other populations.

## 2. Methods

### 2.1. Study Design and Setting

**Figure 1:**
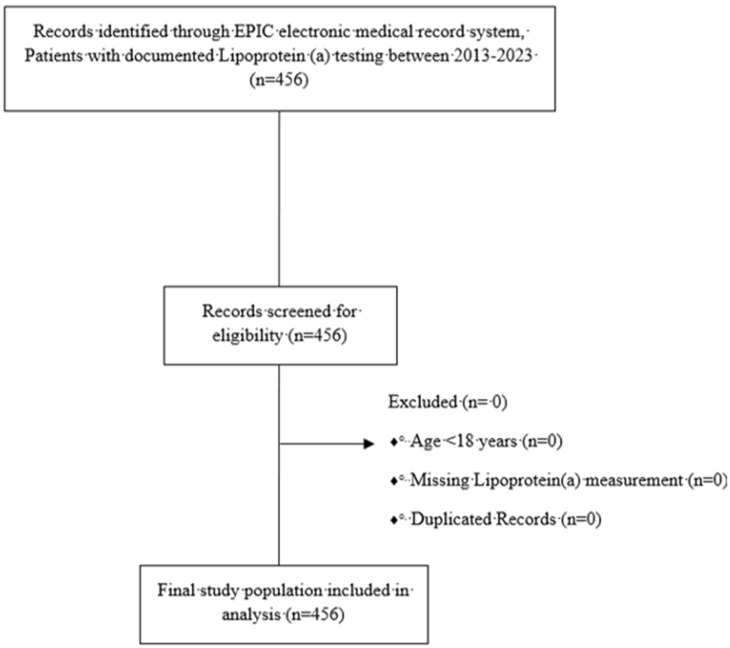
Flowchart depicting the study population selection. A flow diagram illustrating patient identification, eligibility screening, exclusions, and final inclusion in the analytic cohort.

This retrospective observational cohort study was conducted at the AUBMC, the largest tertiary referral center in Lebanon. Patients aged ≥ 18 years who underwent Lp(a) testing at the AUBMC between 2010 and 2023 were included (Figure 1). Exclusion criteria included age < 18 years, absence of a documented Lp(a) level, and duplicate patient records; no duplicate records were identified (Figure 1). The study protocol conformed to the ethical guidelines of the 1975 Declaration of Helsinki and was approved by the AUBMC Institutional Review. Patient consent was waived given its retrospective nature. Patients were classified as male or female based on sex designated at birth. This study was conducted and reported in accordance with the Strengthening the Reporting of Observational Studies in Epidemiology (STROBE) guidelines [13]. 

### 2.2. Data Collection and Management

Clinical and laboratory data of the patients were retrospectively extracted from the EPIC electronic medical record system, a comprehensive, institution-wide digital platform used for clinical documentation and laboratory reporting at AUB. The primary study variable was Lp(a) level, which is measured as part of clinical care for risk prevention and stratification in patients with moderate risk at the AUBMC laboratory using a Cobas Integra 400 Plus analyzer. All biochemical measurements are performed in the same laboratory according to manufacturer instructions and institutional protocols, with routine internal quality control procedures to ensure standardization and accuracy.

Clinical variables included diabetes mellitus (DM); hypertension (HTN); dyslipidemia; dyslipidemia treatment; aspirin use; and levels of vHbA1c, LDL, high-density lipoprotein (HDL), total cholesterol, and triglycerides. Outcomes included cardiovascular history: coronary artery disease (CAD), previous coronary artery bypass grafting (CABG), heart failure (HF), previous myocardial infarction (MI), aortic stenosis (AS), atrial fibrillation (AF), cerebrovascular disease (CVD), peripheral artery disease (PAD). Sociodemographic characteristics (age, sex) and anthropometric measures (weight, height) were collected as covariates.

To minimize information bias inherent to retrospective chart reviews, laboratory values were obtained directly from structured laboratory reports, and clinical diagnoses and comorbidities were extracted from documented physician diagnoses, problem lists, and structured clinical notes. The records extracted were screened for internal consistency and duplicate entries to reduce abstraction errors.

## 3. Statistical Analysis

Statistical analysis was performed using IBM SPSS Statistics for Windows, Version 28.0 (IBM Corp., Armonk, NY, USA). Descriptive statistics summarized the data: categorical variables were expressed as frequencies and percentages, while continuous variables were presented as means and standard deviations. Lp(a) levels were additionally categorized using predefined cutoffs of ≥30, ≥50, and ≥70 mg/dL [14]. Pearson correlation was used to assess the relation between two continuous variables, while logistic regression was used to assess association between an independent continuous variable and a dependent categorical variable. The one-sample T-test was done to compare the mean Lp(a) of our patients to that reported in the United States population [15]. Subgroup analysis by sex was done to study the association between Lp(a) with CAD and AF. In addition, subgroup analysis of Lp(a) > 50 was done to study its association with AF. A *p*-value below 0.05 was set as the threshold for statistical significance.

## 4. Results

**Figure 2:**
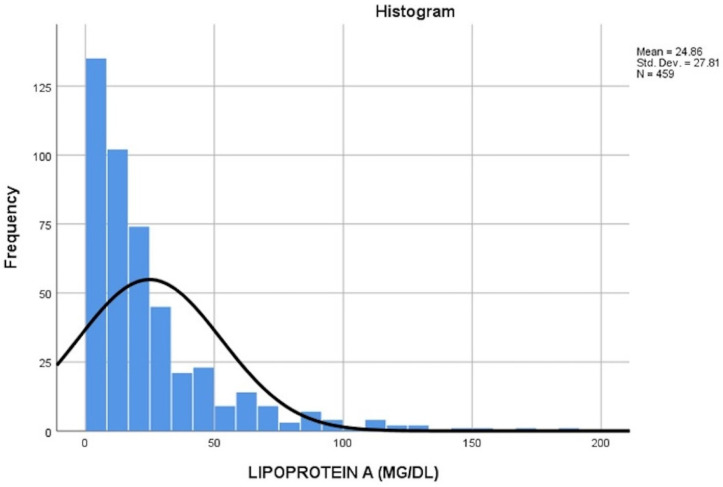
Frequency distribution of Lp(a) in a tertiary center in Lebanon. Histogram showing the frequency distribution of serum Lp(a) mass (mg/dL) level among 459 participants. The distribution is right-skewed, with a mean Lp(a) level of 24.9 mg/dL and SD of 27.8. Lp(a): lipoprotein(a); SD: standard deviation; mg/dL: milligrams per deciliter.

A total of 456 patients with a mean age of 50 ± 13 were included in the study, where males constituted 59%. The mean Lp(a) level was 25 ± 28 mg/dL, and median Lp(a) was 15.5, with a range of 0 to 188 (Figure 2). Lp(a) mass levels were skewed to the right. In total, 119 patients (26%) had Lp(a) levels above 30, while 59 (13%) had Lp(a) levels above 50, and 35 (7.6%) had Lp(a) levels above 70. Subgroup analysis by gender revealed that the mean Lp(a) in females was higher than that in males being 28 ± 32 and 23 ± 25 respectively. The mean weight of the population was 78 ± 17 Kgs. With regard to lipid profile, the LDL, HDL, total cholesterol, and triglyceride means were 111 ± 40, 52 ± 18, 192 ± 24, and 131 ± 97, respectively. Additionally, 41% (175) of the patients had dyslipidemia, while 40% (171) had dyslipidemia treatment. Furthermore, the background medical history was analyzed, revealing that the most prevalent comorbidity among patients was HTN, with a prevalence of 37% (158). Next was DM at 20%. Additionally, 28% (120) of patients were on aspirin, and 80 patients (19%) had CAD, out of which, 22 (5.2%) underwent CABG. Other variables inspected included HF at 2.4%, a history of MI at 2.1%, AS at 4.9%, AF at 5.2%, CVD at 5.5%, and PAD at 2.8% (Table 1).

The one-sample T-test comparing the mean Lp(a) level of the study population to a previously published reference value showed a mean Lp(a) level in our cohort of 25 ± 28 mg/dL, with a mean difference of −9.14 (95% CI: −11.69–−6.59, *p*-value: < 0.001) (Table 2).

Logistic regression analysis showed no significant causation effect of Lp(a) on the categorical clinical variables and outcomes reported including DM, dyslipidemia, HTN, HF, AF, previous MI, CVD, PAD, CAD and previous CABG (Table 3). An almost significant relationship was found in the subgroup analysis by sex between Lp(a) with CAD in males (Exp(B):1.01, sig.: 0.054) and Lp(a) with AF in females (Exp(B):1.022, sig.: 0.055) (Supplemental Table S1). Similarly, no significant Pearson correlation was found between Lp(a) and HbA1c, LDL, HDL, total cholesterol, and triglyceride levels (Table 4). Lastly, the chi-square test subgroup analysis showed a significant relationship between Lp(a) and AF with Lp(a) above 50 (*p*-value: < 0.024) (Supplemental Table S2).

## 5. Discussion

In this retrospective study, the first of its kind in the cadre of the AUBMC, Lebanon, we primarily assessed the distribution of Lp(a) mass levels in a Middle Eastern population and secondarily examined their association with demographic characteristics, clinical variables and outcomes. The importance of these findings is relevant considering the rising rates of mortality reported from cardiovascular diseases with paucity of conventional risk factors [16]. This warrants additional screening and unveiling of possible associated risk factors.

The mean level of Lp(a) reported in our study was 25 ± 28 mg/dL. This falls behind that reported in Blacks and South Asians but is higher than that reported in White ethnicities [3]. Additionally, a significant difference of 9 mg/dL was found between the reported level of Lp(a) in our population and that reported by Varvel et al. [15], the biggest study assessing Lp(a) characteristics in the U.S. population including 532,359 patients. These differences may reflect population-specific genetic variability in Lp(a) isoform distribution, highlighting the need for caution when generalizing findings from Western cohorts to other populations, including those in the Middle East. In addition, Varvel et al. [15] reported a mean level of Lp(a) in females of 37.0 ± 42.7 and of 30.7 ± 36.7 in males. This was similarly observed in our cohort, which showed higher mean Lp(a) level in females (28 ± 32) than in males (23 ± 25). Several biological mechanisms have been proposed to explain this pattern. Although Lp(a) levels are predominantly genetically determined by Lp(a) Kringle IV type 2 number, hormonal influences appear to modulate their expression [17]. Estrogen has been shown to exert a suppressive effect on Lp(a) concentrations, with lower levels observed in premenopausal women and a significant rise following menopause, coinciding with declining estrogen levels [18]. Studies show a 17–27% rise in women after age 50, with a further increase observed in those not receiving hormone replacement therapy [19]. However, as detailed hormonal, reproductive, and genetic data were not available in the present study, these mechanisms remain speculative in our cohort, and the observed sex differences should be interpreted as descriptive rather than mechanistic. In total, 119 (26%) of patients in our cohort had an Lp(a) level of >30 mg/dL, while 59 (13%) had an Lp(a) level of >50 mg/dL. In comparison, Varvel et al. had 35% of study subjects with Lp(a) levels > 30 mg/dL, and 24% had levels > 50 mg/dL. As indicated in their study, the usual upper limit of normal Lp(a) in U.S. laboratories is 30 mg/dL, while the threshold suggested by the EAS Consensus document is 50 mg/dL [15].

In our study, no statistically significant correlations were observed between Lp(a) levels and conventional lipid measures, including total cholesterol, LDL, HDL, or triglycerides. This finding represents a divergent result compared with some prior reports. For instance, a positive correlation between Lp(a) and both non-HDL-C and total cholesterol, but not HDL-C or triglycerides, was found in a study based on a pediatric Lebanese population; the mean Lp(a) reported in that study was 14.4 [20]. The association between Lp(a) and HDL-C, however, remains inconsistent. Some studies suggest an inverse correlation in insulin-resistant populations, while others report a mild positive association with HDL-C [18]. Similarly, Lp(a) also exhibits a variable relationship with triglycerides depending on the population studied. For example, in patients with familial combined hyperlipidemia, elevated Lp(a) levels were correlated with lower triglyceride concentrations, higher HDL-C, and a reduced waist circumference [21]. Research on VLDL-C and non-HDL-C in cardiovascular risk assessment remains limited. Nevertheless, Lp(a) has been identified as an independent contributor to residual cardiovascular risk, suggesting that its role extends beyond traditional lipid markers [22]. Taken together, these findings suggest that the relationship between Lp(a) and traditional lipid parameters is heterogeneous, varies across populations, and is shaped by a combination of genetic, metabolic, and environmental influences. The lack of significant correlations observed in our study should be interpreted as a null finding limited by the small sample size, underpowering, and ethnic attributes rather than as evidence against the independent role of Lp(a) in modulating lipid levels and cardiovascular risk. HTN was the most prevalent comorbidity in our cohort, affecting 158 (37%) patients. No significant association between HTN and Lp(a) level was observed. This goes in line with the results reported by Zheutlin et al., which included 5307 patients, and Ward et al., which included 167 patients [23,24]. Both studies found no association between Lp(a) levels and HTN in the overall cohort. However, Zheutlin et al. found a higher risk of HTN in adults older than 50 years with Lp(a) levels higher than 50 mg/dL [23].

Additionally, no significant correlation between Lp(a) levels and vascular diseases including CAD, CVD, PAD, and previous MI was found. However, an exploratory subgroup analysis suggested a potential association between higher Lp(a) levels and CAD among male patients Notably, a growing body of evidence in Western populations demonstrates a strong association between elevated Lp(a) levels and CAD. For example, in the trial done by O’Toole et al., increased Lp(a) levels were associated with a higher risk of CAD in 1815 participants [25]. However, more studies need to be done in the Mediterranean region to explore the underlying genetic factors. With respect to peripheral vascular disease, Klein et al. reported an association between higher Lp(a) levels and carotid artery sclerosis and occlusion [26]. However, the relationship between Lp(a) and broader peripheral outcomes, including cerebrovascular disease (CVD) and peripheral artery disease (PAD), remains incompletely established and warrants further investigation [27,28]. In addition, a large multiethnic study including seven ethnic groups found weaker associations between elevated Lp(a) and myocardial infarction among Arab and African participants than in other populations [29], which may help explain the absence of a significant association in our cohort. Finally, no statistically significant association was found between higher levels of Lp(a) and AF in the overall cohort. However, subgroup analyses showed a significant relationship between Lp(a) above 50 and AF. Moreover, the association between Lp(a) and AF in females approached statistical significance (Exp(B):1.022, sig.: 0.055). Nonetheless, these findings were based on subgroup analysis and should be interpreted with caution. Compared with the existing literature, Aronis et al. found no association between AF and increasing Lp(a) levels among either Black or White participants, regardless of sex in a large community-based study of 10,127 participants. In contrast, other studies have reported different patterns: Tao et al. observed an inverse association in a retrospective analysis, whereas Mohammadi-Shemirani et al. suggested a positive causal relationship using Mendelian randomization [30,31]. These discrepancies may reflect population-level differences in genetically determined Lp(a) thresholds at which risk becomes clinically relevant, with AF risk potentially emerging at different Lp(a) cutoffs across ethnic groups. From a mechanistic perspective, several biological pathways have been proposed to link elevated Lp(a) levels with AF. Lp(a) was described to have proinflammatory and pro-thrombotic properties and has been associated with endothelial dysfunction and microvascular ischemia, which may contribute to atrial structural remodeling [32,33].

The present findings extend the existing literature by providing population-specific data on Lp(a) levels from a Middle Eastern adult cohort. Middle Eastern population remains underrepresented in current evidence and some diseases are more proportionally found in this population [34]. While elevated Lp(a) is widely recognized as a cardiovascular risk modifier in Western populations, the largely non-significant associations observed in this study highlight the potential limitations of directly extrapolating risk stratification models and screening strategies across populations. From a clinical perspective, these results indicate that the interpretation of Lp(a) measurements in Middle Eastern patients may benefit from the consideration of population-specific context rather than the uniform application of external risk frameworks. Importantly, this study provides region-specific data that may help refine the ongoing efforts to clarify how Lp(a) is integrated into cardiovascular risk assessment and clinical decision-making.

## 6. Limitations

First, this study is limited by its modest sample size (n = 465) and single-center design, which may restrict statistical power, limit representativeness, and contribute to limited event counts for several cardiovascular outcomes. In addition, because Lp(a) testing is not routinely performed in unselected populations, our cohort likely reflects indication-based testing, introducing potential selection bias and limiting the generalizability of the findings. Second, the retrospective design and largely cross-sectional nature of the analysis preclude causal inference and remain susceptible to residual confounding despite adjustment for measured covariates. Third, reliance on manual chart abstraction introduces the possibility of data-entry or extraction errors, which could affect internal validity. Finally, comparisons with Western cohorts (e.g., from the United States) should be interpreted cautiously, as differences in demographics, genetic background, population characteristics, indications for testing, laboratory assays, and diagnostic thresholds may contribute to divergent findings across studies and limit the validity of direct inference.

## 7. Conclusions

In this study, we provide a descriptive characterization of Lp(a) levels in a Lebanese adult population. The clinical importance of this study lies in providing regional data that can enhance the understanding of Lp(a) levels in Middle Eastern adult populations. While a previous study assessing Lp(a) levels in Lebanon focused solely on a pediatric cohort, the present study extends the available data to an adult tertiary-care population, addressing an important regional knowledge gap. In secondary analyses, most associations between Lp(a) levels and cardiovascular or metabolic conditions were not statistically significant. These findings do not exclude potential associations, particularly given the exploratory nature of the analyses and the limited power of the study. Rather, they further highlight the heterogeneity of Lp(a)-related associations reported across different populations. This study underscores the need for larger, prospective studies to better define the clinical significance of Lp(a) levels and their relationship with cardiovascular outcomes in Middle Eastern populations.

## Figures and Tables

**Table 1:** Basic characteristics of the study population (N = 456).

Variable	Mean ± SD
Age	50.2 ± 13.49
Lipoprotein A	24.86 ± 27.81
Weight	77.86 ± 17.15
LDL	110.5 ± 39.42
HDL	51.52 ± 17.73
Total cholesterol	191.78 ± 24.06
Triglyceride	130.66 ± 966.72
HbA1c	5.84 ± 5.14
Lipoprotein A in males	22.97 ± 24.807
Lipoprotein A in females	27.63 ± 31.547
	% (N)
Lipoprotein A levels ≥ 30	25.9% (119)
Lipoprotein A levels ≥ 50	12.9% (59)
Lipoprotein A levels ≥ 70	7.6% (35)
Sex	
Male	59% (269)
Female	41% (187)
Dyslipidemia	41.3% (175)
Dyslipidemia treatment	40.2% (171)
Hypertension	37.1% (158)
Diabetes mellitus	20.2% (85)
On aspirin	28.3% (120)
CAD	19% (80)
Previous CABG	5.2% (22)
Heart failure	2.4% (10)
Previous MI	2.1% (9)
AS	4.9% (11)
AF	5.2% (22)
CVD	5.5% (23)
PAD	2.8% (12)

LDL: low-density lipoprotein; HDL: high-density lipoprotein; CAD: coronary artery disease; CABG: coronary artery bypass graft; MI: myocardial infarction; AS: aortic stenosis; AF: atrial fibrillation; CVD: cerebrovascular disease; PAD: peripheral artery disease.

**Table 2:** One-sample T-test comparing our mean Lp(a) (25 mg/dL) to test value of 34 mg/dL.

	t	df	Sig. (2-Tailed)	Mean Difference	95% Confidence Interval, Lower	95% Confidence Interval, Upper
Lipoprotein A (MG/DL)	−7.045	458	0.000	−9.144	−11.70	−6.59

One-sample T-test was used to compare the mean Lp(a) level of a Lebanese population to that of the U.S. population, computed by Varvel et al. [15].

**Table 3:** Logistic regression of lipoprotein(a) with categorical health variables.

	B	S.E.	Wald	df	Significance	Exp(B)
Dyslipidemia	0.006	0.004	2.360	1	0.124	1.006
Dyslipidemia treatment	0.004	0.004	1.002	1	0.317	1.004
Hypertension	−0.003	0.004	0.788	1	0.375	0.997
DM	−0.005	0.005	0.882	1	0.348	0.995
Aspirin intake	0.001	0.004	0.075	1	0.785	1.001
CAD	0.004	0.004	1.020	1	0.313	1.004
Previous CABG	−0.002	0.009	0.055	1	0.814	0.998
HF	−0.017	0.018	0.835	1	0.361	0.983
Previous MI	0.008	0.010	0.569	1	0.450	1.008
AS	−0.019	0.020	0.928	1	0.335	0.981
AF	0.010	0.006	2.222	1	0.136	1.010
CVD	0.003	0.008	0.120	1	0.729	1.003
PAD	0.001	0.011	0.018	1	0.895	1.001

Logistic regression analysis examining the association between lipoprotein(a) levels and various categorical variables. B: regression coefficients; S.E.: standard error; df: degrees of freedom; Exp (B): odds ratio; DM: diabetes mellitus; CAD: coronary artery disease; CABG: coronary artery bypass graft; HF: heart failure; MI: myocardial infarction; AS: aortic stenosis; AF: atrial fibrillation; CVD: cerebrovascular disease; PAD: peripheral artery disease.

**Table 4:** Pearson correlation of lipoprotein(a) with HbA1c and lipid profile components.

		HbA1c	LDL	HDL	Total Cholesterol	Triglyceride
Lipoprotein A (MG/DL)	Pearson Correlation	−0.029	−0.017	0.005	−0.016	−0.050
	Significance (2-tailed)	0.569	0.734	0.926	0.756	0.315

Pearson correlation evaluating the association between lipoprotein(a) levels and different continuous variables: HbA1c, LDL, HDL, total cholesterol, and triglyceride levels. HbA1c: hemoglobin A1C; LDL: low-density lipoprotein; HDL: high-density lipoprotein.

## Data Availability

The data presented in this study are available on request from the corresponding author.

## Supplementary Materials

The following supporting information can be downloaded at https://www.mdpi.com/article/10.3390/jcm15041461/s1, Table S1: Logistic regression of Lp(a) with CAD and AF stratified by sex; Table S2: Chi-square test of Lp(a) above 50 with atrial fibrillation.
