# Supplementary material for: Distribution of Lipoprotein(a) Levels and Clinical Associations in a Lebanese Adult Population: A Retrospective Observational Study
Source: J Clin Med. 2026 Feb 13;15(4):1461. doi: 10.3390/jcm15041461 (PMC12942122; doi:10.3390/jcm15041461)
Supplement: Supplementary file 1 [file jcm-15-01461-s001.zip › jcm-4087902-supplementary.pdf]

Table S1. Logistic regression of Lp(a) with CAD and AF stratified by sex.

	B	S.E.	Wald	df	Significance	Exp(B)
CAD and LIPOPROTEIN A (MG/DL)						
Sex: Male	0.010	0.005	3.720	1	0.054	1.010
AF and LIPOPROTEIN A (MG/DL)						
Sex: Female	0.022	0.012	3.672	1	0.055	1.022

Logistic regression analysis examining the association between Lp(a) levels and coronary artery disease and atrial fibrillation stratified by male and female sex respectively. B: regression coefficients; S.E.: standard error; df: degrees of freedom; Exp (B): odds ratio; CAD: coronary artery disease; AF: atrial fibrillation.

Table S2. Chi-square test of Lp(a) above 50 with atrial fibrillation.

	Value	df	Significance
Pearson chi square	5.081[a]	1	0.024

df: degrees of freedom.
